# Supplementary material for: Factors Associated with Willingness toward Organ Donation in China: A Nationwide Cross-Sectional Analysis Using a Social–Ecological Framework
Source: Healthcare (Basel). 2023 Mar 10;11(6):824. doi: 10.3390/healthcare11060824 (PMC10048496; doi:10.3390/healthcare11060824)
Supplement: Supplementary file 1 [file healthcare-11-00824-s001.zip › healthcare-2224393-supplementary.pdf]

Supplementary Material

Supplementary Figures and Tables

Table S1. Self-efficacy Questions and Assignment Criteria

Table S2. Personality Questions and Assignment Criteria

Table S3. Health literacy and Assignment Criteria

Table S4. Family Health Questions and Assignment Criteria.

Table S5. Perceived Social Support Questions and Assignment Criteria.

Table S6. Media Use.

Table S1. Self-efficacy Questions and Assignment Criteria

There are 8 sentences below, each followed by 5 answers. Please make your choice in relation to your situation.

1.I will be able to achieve most of the goals I have set for myself.
2.I am sure I can accomplish them when facing with difficult tasks.
3.Overall, I think I can get results that are important to me.
4.I believe I can succeed in anything as long as I make up my mind to it.
5.I will be able to overcome many challenges successfully.
6.I am confident that I can accomplish many different tasks effectively.
7.I can accomplish most tasks well compared with other people.
8.I can perform well even when things are tough.

Questions are positively scored: Strongly disagree =1; Slightly disagree =2; Neither agree nor disagree =3; Slightly agree =4; Strongly agree =5

The total score is the sum of each question, with a minimum score of 8 and a maximum score of 40. Higher scores indicate better self-efficacy.

Table S2. Personality Questions and Assignment Criteria

There are 10 sentences below, each followed by 5 answers. Please make your choice in relation to your situation.

1.I don't think I talk too much.
2.I believe I trust others in general.
3.I suppose I'm lazy.
4.I think I have a strong capability to work under pressure and easy to relax.
5.I don't consider myself very interested in art.
6.I think I am open-minded and have strong social skills.
7. I am a person who likes to pick others' flaws.
8.I believe I always do my work with carefulness and deep consideration.
9.I suppose I tend to be nervous or anxious.
10.I believe I am a person with rich imagination.

Questions are positively scored: Strongly disagree =1; Slightly disagree =2; Neither agree nor disagree =3; Slightly agree =4; Strongly agree =5.

Table S3. Health literacy and Assignment Criteria

There are 12 sentences below, each followed by 4 answers. Please choose one answer after each sentence according to how easy or difficult the following acts are for you.

1.Can you find information on treatments of illnesses that concerning you?
2.Can you understand the instructions that come with your medicine?
3.Can you judge the advantages and disadvantages of different treatment options?
4.Can you call an ambulance in an emergency?
5.Can you find information on how to manage mental health problems like stress or depression?
6.Can you understand why you need health screenings (such as breast exam, blood sugar test, blood pressure)?
7.Can you tell which vaccinations you may need?
8.Can you decide how to protect yourself from illness based on advice from your family or friends?
9.Do you think activities (such as meditation, exercise, walking, Pilates etc.) that are good for your mental well-being?
10.Is it easy for you to understand information from media (such as Internet, newspaper, magazines) on how to get healthier?
11.Can you judge which daily behavior (such as drinking,eating habits and exercise etc.) is related to your health?
12.Join a sports club or exercise class if you want to?

Very difficult=1; Difficult=2; Easy=3; Very easy=4.

The total score is the sum of each question, with a minimum score of 12 and a maximum score of 48. Higher scores indicate higher health literacy .

Table S4. Family Health Questions and Assignment Criteria.

There are 10 sentences below, each followed by 5 answers. Please make your choice in relation to your situation.

1.We support each other.
2.I feel safe in my family relationships.
3.We help each other in seeking health care services when needed (such as making doctor's appointments).
4.We help each other in making healthy changes.
5.We are full of hope even in difficult times.
6.We do not trust doctors or other health professionals (R).
7.When we have problems at work or school we can turn to people other than our family for help.
8.We have people other than family members who can turn to for a loan (e.g.for $200) if we need financial help.
9.My family did not have enough money at the end of the month after bills were paid (R).
10.My family did not have adequate housing (R).

Questions 1, 2, 3, 4, 5, 7 and 8 are positively scored: Strongly disagree =1; Slightly disagree =2; Neither agree nor disagree =3; Slightly agree =4; Strongly agree =5

Questions 6, 9 and 10 are negatively scored: Strongly disagree =5; Slightly disagree =4; Neither agree nor disagree =3; Slightly agree =2; Strongly agree =1

The total score is the sum of each question, with a minimum score of 10 and a maximum score of 50. Higher score indicates better family health index.

Table S5. Perceived Social Support Questions and Assignment Criteria.

There are 12 sentences below, each followed by 7 answers. Please choose one answer after each sentence according to your actual situation.

1.People (relatives, neighbors, colleagues) always be there for me when I have problems.
2.I can share my joys and sorrows with some people (relatives, neighbors, colleagues).
3.My family is able to solve my problems in a practical way.
4.I can get emotional help and support from my family when I need it.
5.There are people (relatives, neighbors, colleagues) who are a real source of comfort when I am in trouble.
6.My friends can really help me.
7.I can rely on my friends in times of difficulty.
8.I can talk about my problems with my family members.
9.My friends can share my happiness and sadness with me.
10.There are people in my life (relatives, neighbors, colleagues) who care about my feelings.
11.My family members are willing to assist me in making decisions.
12.I can talk about my problems with my friends.

Extremely disagree=1； Strongly disagree =2； Slightly disagree=3； Neutral=4

Slightly agree=5； Strongly agree=6； Extremely agree=7.

Table S6. Media Use.

There are 7 media use items in the table and 5 answers for frequency of use. Please choose one answer for each item based on your actual situation.

1.Newspaper
2.Magazines
3.Broadcast
4.TV
5.Books (non-textbook)
6.Personal computers (including tablets)
7.Smartphones

Never use =0; Occasionally use=1; Sometimes use =2; Often use =3; Almost daily= 4.

The total score is the sum of each question, with a minimum score of 0 and a maximum score of 28. Higher score indicates better media use.
